# A method for model-free partial volume correction in oncological PET

**DOI:** 10.1186/2191-219X-2-16

**Published:** 2012-04-24

**Authors:** Frank Hofheinz, Jens Langner, Jan Petr, Bettina Beuthien-Baumann, Liane Oehme, Jörg Steinbach, Jörg Kotzerke, Jörg van den Hoff

**Affiliations:** 1 PET Centre, Institute of Radiopharmacy, Helmholtz-Zentrum Dresden-Rossendorf, Dresden, Germany; 2 Department of Nuclear Medicine, University Hospital Carl Gustav Carus, Technische Universität Dresden, Dresden, Germany

**Keywords:** Partial volume effect, Partial volume correction, Recovery correction, PET, Quantification

## Abstract

**Background:**

As is well known, limited spatial resolution leads to partial volume effects (PVE) and consequently to limited signal recovery. Determination of the mean activity concentration of a target structure is thus compromised even at target sizes much larger than the reconstructed spatial resolution. This leads to serious size-dependent underestimates of true signal intensity in hot spot imaging. For quantitative PET in general and in the context of therapy assessment in particular it is, therefore, mandatory to perform an adequate partial volume correction (PVC). The goal of our work was to develop and to validate a model-free PVC algorithm for hot spot imaging.

**Methods:**

The algorithm proceeds in two automated steps. Step 1: estimation of the actual object boundary with a threshold based method and determination of the total activity A measured within the enclosed volume V. Step 2: determination of the activity fraction B, which is measured outside the object due to the partial volume effect (spill-out). The PVE corrected mean value is then given by *C*_mean_ = (A+B)/V. For validation simulated tumours were used which were derived from real patient data (liver metastases of a colorectal carcinoma and head and neck cancer, respectively). The simulated tumours have characteristics (regarding tumour shape, contrast, noise, etc.) which are very similar to those of the underlying patient data, but the boundaries and tracer accumulation are exactly known. The PVE corrected mean values of 37 simulated tumours were determined and compared with the true mean values.

**Results:**

For the investigated simulated data the proposed approach yields PVE corrected mean values which agree very well with the true values (mean deviation (± s.d.): (−0.8±2.5)*%*).

**Conclusions:**

The described method enables accurate quantitative partial volume correction in oncological hot spot imaging.

## Background

In recent years PET has become more and more important for therapy response assessment in oncology. In this context quantitation has been mostly restricted to assessment of changes of the maximum standardised uptake value (SUV_max_) of lesions during therapy [1], but there are also attempts to correlate the SUV_mean_ of lesions with therapy outcome, which might be a more representative parameter especially for lesions with heterogeneous tracer accumulation (see e.g. [2,3]). However, the limited spatial resolution of PET leads to partial volume effects PVE and, consequently, to limited signal recovery for, both, SUV_*max*_ and SUV_*mean*_. While SUV_max_ is affected only for small structures (whose size is comparable to – or smaller than – the given spatial resolution), SUV_mean_ is compromised even at target sizes much larger than the reconstructed spatial resolution [4,5]. Therefore, it is mandatory to perform an adequate PVE correction.

There exist several strategies for PVE correction (see[6-8] for recent reviews). Most often the PVE correction is computed on the basis of phantom measurements, where the signal recovery is determined for different object sizes and different background values (see e.g. [9-13]). The PVE correction is then performed using the signal recovery of a phantom with approximately the same volume and background as the target structure. Another approach is to improve spatial resolution either via deconvolution of the reconstructed PET data [14-19] or via integrating partial volume correction into the image reconstruction [18,20-25]. A different strategy is to use model-free correction schemes, which directly determine the spill-out from the target structure but require knowledge of the object’s boundary and its background [26-28]. However, although many approaches have been shown to work in principle, there exists till now no general consensus regarding the best algorithm to use. Moreover, most algorithms are not generally available, neither in the public domain nor in commercial tools.

In this paper we present a model-free method for PVE correction of the SUV_mean_ of focal structures. Our method can be considered as an extension of the methods reported in [26,28]. In these papers, the object boundaries are determined in CT data and the mean value of a separate background ROI is considered as representative of the actual background of the target structure. Our extension is twofold: first, the object boundaries are determined directly in the PET data and second, for each voxel in the spill-out region a local background is computed independently instead of using a common background value for the complete ROI. For validation of the proposed approach the method was applied to simulated lesions, which were generated from (and embedded in) actual clinical patient data sets. The resulting “anthropomorphic digital phantoms” provide much more realistic conditions than conventional phantom measurements (which typically use regular shapes and homogeneous tracer distributions in target and background) and are visually not distinguishable from actual patient data. In the absence of a real gold standard we regard this as the best approach to evaluation of our algorithm.

## Materials and methods

### Partial volume effect

**Figure 1:**
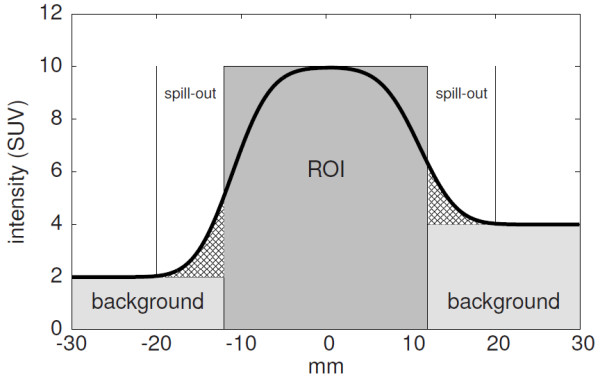
**Illustration of the partial volume effect.** A central cross-section through a homogeneous sphere is shown. The dark grey area represents the sphere and the light grey areas indicate the surrounding background. The thick line is the measured signal at finite spatial resolution.

The partial volume effect is illustrated in Figure 1. The dark grey area represents a homogeneous sphere with diameter 24 mm in a hot background (light grey). In general the background is not homogeneous, which is here exemplified by different background levels on the left and right side of the sphere. The thick black line represents the measured signal at a spatial resolution of 8 mm (here and in the following specified as full width at half maximum (FWHM) of the corresponding point spread function). The shaded areas represent the effective spill-out from the ROI (difference of spill-out from the ROI and spill-in from the surrounding background). The spill-out activity, *A*_sp_, is not measured within the true object boundary (presuming this is known) and, therefore, the mean concentration, *C*_mean_, is underestimated. If the true object boundary is known, the activity *A*_sp_ can be computed by summing up the background corrected activity of voxels which are inside the spill-out region. This was already proposed in [26,28], where the background is approximated by the average of a 2D or 3D region in the vicinity of the target structure. We extend this method to a local background, which is computed independently for each voxel in the spill-out region. *A*_sp_ is then given by

(1) $$A_{\mathrm{sp}}=V_{\mathrm{vox}}\underset{v\in \mathrm{sp}}{\sum }\left(C(v)-B(v)\right)\;,$$

where summation is performed over all voxels in the spill-out region, *V *_vox_ is the volume of a single voxel, *C*(*v*) the measured activity concentration of voxel *v* and *B*(*v*) the corresponding background concentration. The PVE corrected mean concentration of the ROI, $C_{\mathrm{mean}}^{\;\mathrm{corr}}$, is then computed as

(2) $$C_{\mathrm{mean}}^{\;\mathrm{corr}}=\frac{A_{\mathrm{ROI}}+A_{\mathrm{sp}}}{V_{\mathrm{ROI}}}\;\mathrm{with}\;A_{\mathrm{ROI}}=C_{\mathrm{mean}}V_{\mathrm{ROI}}\;,$$

where *V*_ROI_ is the volume of the ROI. Normalising $C_{\mathrm{mean}}^{\;\mathrm{corr}}$ to the injected activity and the patient weight leads to the PVE corrected SUV_mean_.

#### Algorithm

Equation (2) is solved in two steps: 

1. The boundaries of the ROI are determined using the automatic ROI delineation method implemented in ROVER, ABX, Radeberg, Germany, which uses adaptive thresholding for ROI delineation (see [29] for details). The delineation also provides *C*_mean_ and *V*_ROI_.

2. After ROI delineation the spill-out region is identified and for each voxel inside the spill-out region the local background is computed (see below). Then *A*_sp_ is calculated according to Eq. (1).

Inserting the results from 1. and 2. in Eq. (2) leads to the PVE corrected mean value. In the following we refer to this algorithm as local background partial volume correction (LBPVC ). For comparison, we also compute the PVE corrected mean value using a global background for each ROI (see below). This algorithm is referred to as global background partial volume correction (GBPVC ).

#### Local background

**Figure 2:**
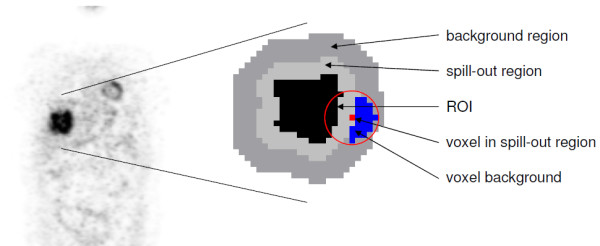
2D illustration of the determination of the local background in spill-out region.

After the initial ROI delineation, the spill-out region and corresponding background region have to be defined. The extent of the spill-out region is obviously dependent on the spatial resolution of the given image data. We take this fact into account by defining the spill-out region as the set of all voxels whose 3D distance *d* to the ROI boundary obeys *d*≤ FWHM (light grey in Figure 2). At this distance the signal has already dropped effectively to the given background level (to a value of about 0.5% of the background corrected true mean concentration in the case of a homogeneous sphere) which thus limits the extent of the spill-out region. In the second step, the background region is defined by determining all voxels with FWHM <*d*≤2.5· FWHM (dark grey in Figure 2). This range corresponds to a shell with a thickness of 1.5· FWHM (about 8-12 mm for typical values of the reconstructed resolution) and simultaneously ensures a sufficiently localised background region as well as a size adequate for obtaining sufficient statistical accuracy. The local background of a single voxel inside the spill-out region is determined by analysing only background voxels in the close vicinity of the voxel as follows. The local background for each voxel in the spill-out region is computed as the average of all surrounding voxels whose distance to the current voxel (red spot in Figure 2) fulfills *d*≤1.5· FWHM (red circle in Figure 2) and which are actually belonging to the background region (blue in Figure 2). As can be seen, the search range is adjusted in such a way that it matches the width of the background region. Moreover, neighbouring ROIs (if present) as well as their spill-out regions are excluded from background determination. If none of the surrounding voxels within the search range belongs to the background region (as can happen, e.g., for voxels in a central necrosis of a tumour), the background of the affected target voxel is assumed to be equal to the average of the complete background region. Note that the spatial resolution, which is usually not known exactly, is entering only in the definition of the boundaries of spill-out and background region (that, moreover, have to be rounded to the nearest neighbouring voxel): the estimated resolution value is affecting only the above-mentioned range conditions. In our case, the estimated spatial resolution is 8 mm (see below), which for a voxel size of 4 mm leads to a thickness of the spill-out and background shells of 2 and 3 voxels, respectively.

The algorithm operates completely in 3D and runs fully automatic after ROI delineation. The computation time is less than one second (including volume delineation) per ROI on an AMD Opteron Processor (model 8356, 2.3 GHz).

#### Global background

The global background method uses a common background value for each ROI. The background region is defined in the same way as for the local background method. The global background value is then computed as the average value of the entire background region. Neighbouring ROIs as well as their spill-out regions are excluded from background determination. Note that this global background is different from the global background used in [26-28]: it is always computed in a matching background shell around the respective ROI even for irregular shaped ROIs and should, thus, provide a more realistic estimate of the actual average background of the given ROI.

### Simulated data

The method was validated on simulated data. In such data the target structures have well known boundaries and tracer accumulation. The simulated data where created by modifications to a number of clinical PET data sets.

#### Study sample

**Figure 3:**
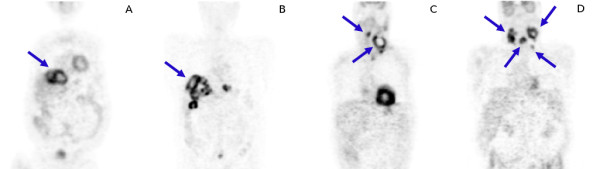
Representative coronal slices for two patients with liver metastases of a colorectal carcinoma (A, B) and for two patients with head and neck cancer (C, D).

The investigated heterogeneous patient group included 13 subjects (8 men and 5 women, mean age 60 years, range 37-79), 5 subjects with liver metastases of a colorectal carcinoma, 8 subjects with head and neck cancer. All subjects underwent a whole-body FDG-PET scan. The PET scans were performed with an ECAT EXACT HR^+^, Siemens/CTI, Knoxville, Tennessee (3D acquisition, 8 min emission and 4 min transmission per bed position). Data acquisition started 1 hour after injection (270 - 370 MBq FDG). Tomographic images were reconstructed using attenuation weighted OSEM reconstruction (6 iterations, 16 subsets, 6 mm FWHM Gaussian filter). The target structures (tumours/metastases) in the patient group had approximate volumes between 3 mL and 500 mL. Lesions with diameter <2· FWHM were excluded from evaluation. Altogether 37 target structures were evaluated. Figure 3 shows representative coronal slices from different patient investigations. The blue arrows indicate the evaluated lesions.

#### Simulation procedure

**Figure 4:**
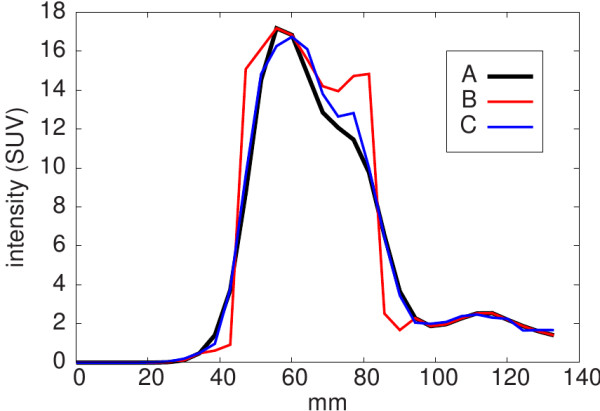
**Line profiles through the tumour shown in Figure **5**.A**: original data. **B**: simulated data before smoothing. **C**: simulated data after smoothing.

In the first step the lesions in the patient data were delineated using the above mentioned delineation algorithm. In each case, the resulting boundary was used as the true boundary of a new, simulated lesion. Corresponding individual spill-out and background regions were then determined as described above. In the second step the intensity value of each voxel in the spill-out region was replaced by the value of its local background derived from the blue region in Figure 2. The difference of the old (true) value and the new one (local background), *C*(*v*)−*B*(*v*), was then distributed equally over all object voxels within the red circle (i.e., intersection of red circle with black area in Figure 2). This corresponds to an approximate compensation of the spill-out (and maintains the total activity of the target structure). Both modifications together lead to a very sharp edge at the object boundary, see the red curve in Figure 4. The shape of the resulting simulated lesion is very similar to that of the original one, but the sharp boundary unambiguously defines the true volume of (and activity distribution within) this target structure. In a final step the simulated structure is smoothed with a 8 mm FWHM Gaussian filter and adequate Gaussian noise is added to the data to mimic the underlying patient data as closely as possible. A FWHM of the Gaussian noise distribution of 5% (relative to the given voxel intensity) most closely corresponds to the noise level observed in the actual patient data. This value was therefore adopted throughout the simulations.

**Figure 5:**
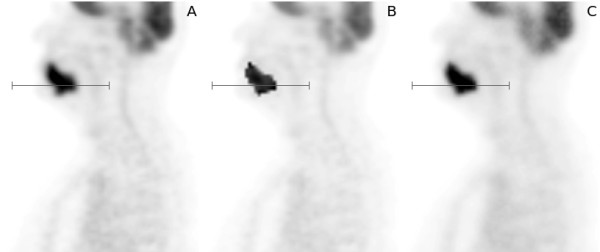
**Illustration of the simulation procedure. A**: original data. **B**: simulated data before smoothing. **C**: simulated data after smoothing. The grey line segment indicates position of the line profiles shown in Figure 4.

The simulated data before smoothing serve as our gold standard for which the true object boundaries, volumes, and SUV_mean_ values are precisely known. In the following, we refer to these values as the true values. The Gaussian filter, applied to these data, then corresponds to an isotropic Gaussian point spread function with FWHM = 8 mm, which leads to approximately the same spatial resolution as in the original image data and, therefore, causes partial volume effects which are very similar to what is happening in real patient data.

Performance of the simulation procedure is illustrated in Figure 5. The original data are shown on the left. In the middle the resulting simulated artificial tumour with sharp boundary is shown, which serves as “ground truth” during evaluation of the algorithm. On the right the same structure after smoothing is shown which represents the “imaged” tumour, for which the PVC is to be evaluated. Figure 4 shows line profiles through the tumour along the grey lines indicated in Figure 5. The described simulation procedure leads to target structures which are very similar to their original counterparts with regard to several parameters such as mean and maximum uptake, target/background contrast, background characteristics, and the degree of heterogeneity (estimated as standard deviation of the mean value).

### Simulation of low contrast structures and variable noise

In the chosen patient group, the simulated lesions obtained with the procedure described above exhibit contrasts (defined as ratio of maximum value to mean background) between 4.0 and 13.1, reflecting the actually observed conditions in this patient group. In order to study the influence of lower contrasts on LBPVC we reduced the voxel values inside the lesion by a factor of two for a subgroup of 5 selected lesions (while keeping surrounding voxels unmodified). In order to avoid secondary problems related to potential merging of lesion and nearby hot spots during the ensuing smoothing step only lesions without further hot spots in the immediate vicinity were selected for this procedure. The resulting additional 5 simulated lesions exhibited contrasts between 2.7 and 3.4. They were further analysed together with the initially simulated lesions in order to determine the contrast dependency of the partial volume correction procedure.We also performed the lesion simulation at three additional noise levels with a FWHM of the Gaussian noise of 3.5%, 7.1%, and 10%, respectively, augmenting the results obtained for FWHM = 5%. The additional noise levels were investigated in order to assess the noise sensitivity of the algorithm. This is of practical relevance, e.g., if scan times and injected doses are modified: in our case, decreasing the noise amplitude by a factor of $\sqrt{2}$ to FWHM = 3.5% is equivalent to a doubling of the scan duration (or injected dose). Accordingly, increasing the noise amplitude by a factor of $\sqrt{2}$ (2) to FWHM = 7.1% (10%) corresponds to reduction of the scan duration by a factor of 2 (4).

### Image analysis

For the simulated data the PVE corrected SUV_mean_ of the ROIs were determined using the LBPVC and GBPVC algorithms, respectively. The values were first computed using the known object boundaries (i.e., omitting the volume delineation step) and second by applying the complete correction scheme including volume delineation. In both cases, the corrected SUV_mean_ was compared with the true SUV_mean_. Moreover, the automatically delineated ROI volumes were compared with the true volumes. For definition of the background and spill-out regions, we used a resolution value of FWHM = 8 mm. In order to test stability of the algorithm against uncertainties of the assumed resolution, we also performed evaluations with spill-out and background regions resulting from assuming resolution values of FWHM = 4 mm and FWHM = 12 mm, respectively. LBPVC and GBPVC correction was performed with the software ROVER (ABX GmbH, Radeberg, Germany).

## Results

### Recovery correction using the true object boundaries

**Figure 6:**
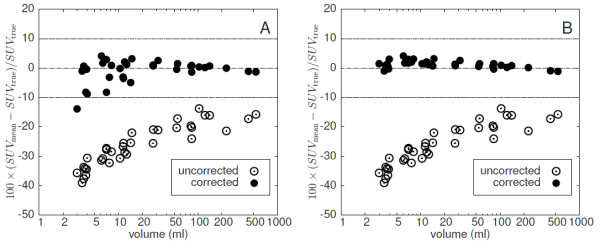
**Deviation of GBPVC-corrected (A) and LBPVC-corrected (B) SUV**_**mean**_**from the true SUV**_**mean**_**using the true object boundaries (at a noise level of FWHM = 5%).** The Uncorrected SUV_mean_ is shown for comparison. Note the logarithmic scale of the abscissa.

Figure 6 shows the fractional deviation of uncorrected and corrected SUV_mean_ from the known true value for GBPVC (A) and LBPVC (B) using the true object boundaries. As expected, the uncorrected data clearly exhibit a reduced mean recovery (down to a recovery coefficient of about 0.6 for the 3 mL lesions). The reduction is size dependent and affects even the largest investigated target structures with volumes of about 500 mL. LBPVC works very good in this case with a deviation of the SUV_mean_ from the true values of (1.2±1.2) %. Accuracy of GBPVC is comparable to LBPVC for large structures but distinctly inferior for smaller ones with volumes below 20 mL. On average the deviation from the true values is (−1.0±3.8) % for GBPVC.

### Recovery correction with automatic ROI delineation

**Figure 7:**
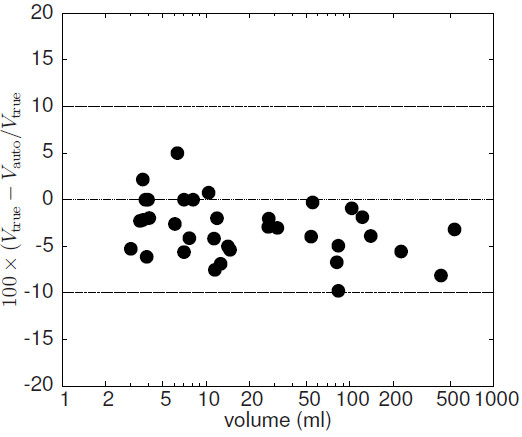
**Fractional deviation of the automatically determined object volumes from the true values (at a noise level of FWHM = 5%).** Note the logarithmic scale of the abscissa.

**Figure 8:**
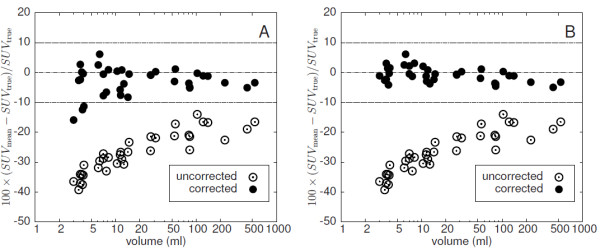
**Deviation of GBPVC-corrected (A) and LBPVC-corrected (B) SUV**_**mean**_**from the true SUV**_**mean**_**using automatic ROI delineation (at a noise level of FWHM = 5%).** The uncorrected SUV_mean_ is shown for comparison. Note the logarithmic scale of the abscissa.

The results of the automatic volume delineation are presented in Figure 7 where the fractional deviation of the measured volumes from the true volumes is shown. As can be seen, the derived volumes underestimate slightly the true volumes (by (−3.2±3.1)%). The deviation remains below 10 % in all cases. Figure 8 shows the fractional deviation of uncorrected and corrected SUV_mean_ from its true value for GBPVC (A) and LBPVC (B), respectively, when performing automatic volume delineation. The deviation of the LBPVC-corrected SUV_mean_ from the true SUV_mean_ always remains below 10% and equals, on average, (−0.8±2.5) *%*. Here, too, GBPVC leads to distinctly larger errors for small target structures, while the deviation for larger objects is comparable to the deviation observed with LBPVC. On average the deviation is (−2.9±4.5) *%*.

### Contrast and noise level dependency

**Figure 9:**
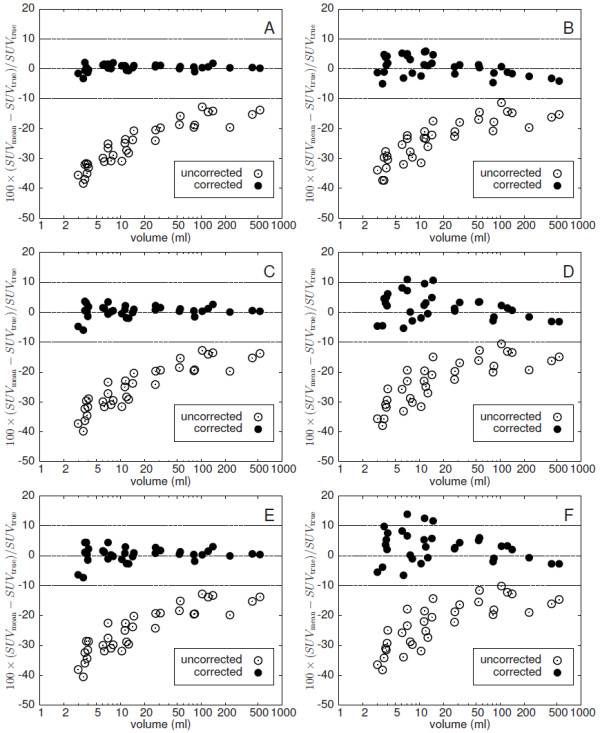
**Difference between LBPVC-corrected and true SUV**_**mean**_**at additional noise levels with a FWHM of the Gaussian noise of 3.5% (A, B), 7.1% (C, D) and 10% (E, F), respectively.** Left column: without automatic ROI delineation (true lesion boundary is used). Right column: with automatic ROI delineation. Note the logarithmic scale of the abscissa

**Figure 10:**
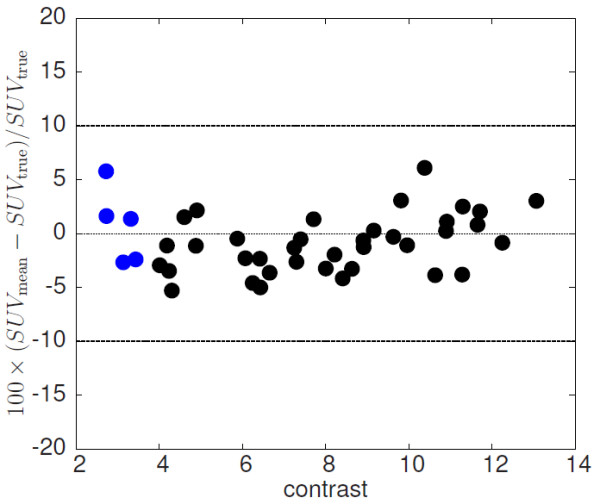
**Contrast dependence of difference between LBPVC-corrected and true SUV**_**mean**_**.** The blue points represent the subgroup of lesions with artificially reduced contrast

The results of LBPVC for the three investigated additional noise levels are shown in Figure 9. In the left column the true ROI boundaries are used for correction, in the right column automatic ROI delineation is used. Reduced noise (A, B) does not lead to substantially improved results in comparison to Figures 6B and 8B, respectively: on average, the deviation of uncorrected and corrected SUV_mean_ from its true value is (0.4 ±1.1) % (A) and (0.7 ± 3.2) % (B), respectively. Elevated noise increases fluctuations of the deviations only slightly when using the true object boundaries ((0.4 ± 2.0) % (C) and (0.4 ± 2.6) % (E), respectively). On the other hand, LBPVC using the automatic delineation leads to notable noise dependent deviations. However, the deviations always remain below 15% ((1.9 ± 4.4) % (D) and (2.9 ± 5.1) % (F) on average, respectively). Figure 10 shows the contrast dependence of the difference between corrected and true SUV_mean_. The blue points represent the additional simulated lesions with an artificially reduced contrast (as described above). The investigated data do not show a systematic dependency on the contrast. Including the additional simulated lesions the deviation of the corrected SUV_mean_ from the true value is on average (0.8 ± 2.7) %.

### Variation of the assumed spatial resolution

**Figure 11:**
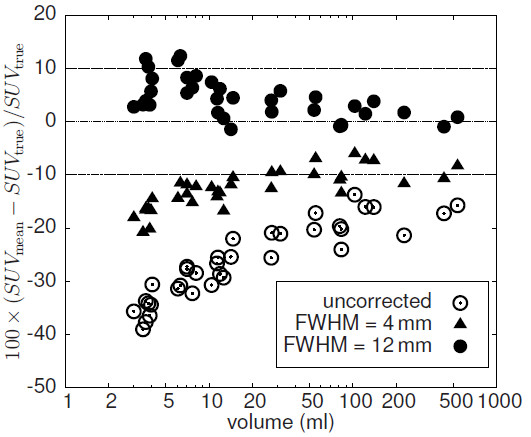
**Difference between LBPVC-corrected and true SUV**_**mean**_**using automatic ROI delineation for different values of the assumed spatial resolution (at a noise level of FWHM = 5%).** Note the logarithmic scale of the abscissa.

Figure 11 shows results of LBPVC for two different values of the assumed FWHM which leads to differently sized spill-out and background regions: FWHM=4 mm (width of spill-out (background) shell: 1 (2) voxels) and FWHM=12 mm (width of spill-out (background) shell: 3 (5) voxels). Processing the data with an assumed resolution of 4 mm leads to systematic underestimates ((−12±3.6)*%*) of the necessary PVE correction. Using FWHM=12 mm leads to a slight overestimate ((4.3±3.8) *%*) of the PVE correction which becomes more distinct for small ROIs.

## Discussion

In this paper we present a model-free method for PVE correction of hot focal structures in PET. We have validated this method using realistic software phantoms of lesions generated from clinical data. The simulated lesions exhibit properties very similar to those of the underlying clinical data sets with respect to relevant parameters (shape/size, contrast, noise, etc.), while having precisely known boundaries and tracer accumulation. The simulated data allowed a direct comparison of the PVE corrected SUV_mean_ resulting from automatic ROI delineation and application of LBPVC with the true SUV_mean_ of the respective simulated lesions. We observe only a small difference between PVE corrected and true values (well below 10% in all cases, mean ± s.d.: (−0.8±2.5) *%*). This high accuracy is achieved by computing a local background for each voxel within a spill-out shell around the automatically delineated ROI. As is obvious from Figure 8, this approach (LBPVC) is superior to application of a common background value (GBPVC) especially for smaller objects with volumes below about 20 mL, even if the common background is determined in a matched background shell around respective ROI (as has be done here). The algorithm depends on a reasonable definition of a spill-out shell which contains all voxels whose activity values are elevated due to spill-out from the ROI plus a background shell whose voxels are assumed to be free of any spill-over effects. We have defined these shells in relative units using the estimated spatial resolution FWHM as the relevant length unit. Therefore, the assumed resolution does have a certain influence on the accuracy of the correction as demonstrated in Figure 11. If the estimate of FWHM is reduced by a factor of two, the necessary PVE correction is underestimated. This is explained by the fact that the spill-out region becomes too small and not all actually affected voxels are included. In this case, the procedure is therefore not able to collect the complete spill-out signal and, consequently, the correction is too small (especially for small structures).

On the other hand, an increase of the FWHM estimate by a factor of 1.5 results only in a slight overcorrection of the actual partial volume effect and the results are quite similar to those obtained with a realistic FWHM value (deviation from true value (4.3±3.8) *%*). Only for 4 out of 37 ROIs the deviation was larger than 10 % (but remained below 15 %) if the too large FWHM was adopted. It can thus be stated that the presented method leads to accurate results as long as the actual FWHM is not substantially underestimated. Accurate knowledge of the spatial resolution is not necessary, however. This is in contrast to e.g. deconvolution techniques [14-19], were the estimated resolution strongly influences the PVE correction. When in doubt (no accurate knowledge of actual spatial resolution), the best strategy, therefore, is to use a pessimistic (i.e. probably too high) estimate for FWHM, e.g. 8 mm even if actual resolution might be 6 mm.

Our method is similar to the methods discussed in [26,28] with the important difference that our background approximation is local. This means the contribution of each voxel in the spill-out region to the PVE correction is computed using the background only in its immediate vicinity (up to a distance of 1.5· FWHM). In this way the method overcomes a limitation of the above mentioned methods which assume a homogeneous background for the whole ROI. In our approach we account for spatial variations of background intensity by determining an individual background level for each voxel and only assume that the background is homogeneous in the very small background area assigned to the respective voxel (blue area in Figure 2). This is a much weaker and more realistic assumption for most clinical PET studies. The superiority of LBPVC over GBPVC can be seen by comparison of Figure 8 (A) and (B). GBPVC (A) leads to reasonable results only for large ROIs (>20 mL). However, the corrected SUV_mean_ of some of the small ROIs substantially deviate from the true values and ROI-to-ROI fluctuation is much higher than with LBPVC .

The proposed PVE correction critically depends on a sufficiently accurate estimate of the true object boundaries (without such an estimate, specification of SUV_mean_ would not make sense anyway). For this task we used a threshold based automatic ROI delineation (see [29]). With this method we achieved good estimations of the true volume (deviation <10 *%*). The observed small deviations of the PVE corrected SUV_mean_ from the true values are essentially an effect of the residual errors in the volume determination (or, rather, boundary delineation) alone. This is demonstrated by using the true object boundaries instead which is possible with our simulated target structures. In this case the difference between corrected and true values is nearly zero (see Figure 6B) which proves that the algorithm is able to correctly estimate the spill-over contributions from all voxels if the true boundary is known.

This shows, that accuracy of the presented PVE correction is essentially limited only by limitations of the used volume delineation process. Difficulties can, therefore, be expected especially for very small objects with diameter <2 · FWHM [4,5]. In this case correct delineation and, therefore, reliable PVE correction method will certainly fail. A second limitation is the degree of heterogeneity in tracer uptake. Heavily heterogeneous ROIs cannot be delineated correctly with threshold based algorithms [30]. The small but systematic underestimation of the delineated ROI volumes shown in Figure 7 can be attributed to this effect. In the present study the heterogeneities of the lesions were moderate (coefficient of intensity variance of voxels within the lesions: 0.13 to 0.22) and the errors in ROI delineation were very small, but it is clear that beyond a certain degree of heterogeneity the method will fail (although such problems can be expected in only a small percentage of the practically relevant cases). Further investigations are necessary to investigate the influence of larger heterogeneities in more detail.

Another factor principally limiting the accuracy of the volume delineation (and of the partial volume correction as well) is a too low contrast of the lesion. However, in our data, covering a contrast range from 2.7 to 13.1, we did not see a clear contrast dependency of the LBPVC correction as demonstrated in Figure 10. Nevertheless, we know from our experience in other investigations that the used delineation algorithm rapidly becomes unstable if the contrast falls below 2.5. Therefore, the presented correction method will not work reliable for such lesions.

A further factor influencing the accuracy of LBPVC is the noise level of the image data as demonstrated in Figure 9. This noise dependency is mainly a consequence of decreased accuracy of the volume delineation at elevated noise levels. Still, we found that the accuracy remains acceptable even if the noise level is doubled (corresponding to a fourfold decrease of scan time in comparison to our standard acquisition protocol): in this case only in 3 out of 37 lesions the error exceeds 10% (while remaining below 15%). It is obvious, however, that in the presence of excessive noise (e.g. in single gates from respiration triggered investigations) the presented correction method would not work reliably. Since the proposed correction algorithm does not require application of the specific delineation method used in this investigation, it could also be combined with alternative delineation algorithms with possibly improved performance (notably for heterogeneous structures). The correction method could of course also make use of available morphological information from CT or MRI for very small lesions or lesions with very low contrast, if available.

All investigated lesions with volumes in the range of 3 to 500 mL exhibited substantially reduced mean (as opposed to maximum) signal recovery. That this is the case even for large lesions is explained by the fact that the partial volume effect is a surface effect and the necessary PVE correction (regarding SUV_mean_) remains sizable even for rather large target structures. The partial volume effect is further increased for irregular/convoluted shapes (compared to approximately spherical objects of the same volume). Irregular shapes are of course not restricted to large structures. In our study sample most lesions with volumes > 10 mL were of distinctly irregular shape (see Figure 3). We consider the ability to perform accurate PVE correction for such structures as the most important benefit of the presented algorithm.

For the validation of our method we used simulated target structures which were derived from clinical data. The simulated target structures are much closer to real clinical data than typical (hardware or digital) phantoms. Realistic regional heterogeneities in the target structure or the background are especially difficult to realise (if at all) with the usual phantoms. The same is true regarding the generation/investigation of irregular shapes. Moreover, the standard spherical phantom inserts are hollow glass spheres whose cold walls can have a strong influence on the measured partial volume effects [31,32] which are, therefore, not representative for the conditions found in real data. All these problems are avoided by our simulation procedure. For example, although the original tumour uptake heterogeneities are indeed modified by the smoothing applied during the simulation procedure (see above), they remain on a realistic level (see profiles in Figure 4). Despite absence of a true gold standard we believe that the performed validation allows to conclude that the proposed algorithm does provide a means for a quite accurate partial volume correction of real patient data.

The accuracy of the partial volume correction achieved in this study is comparable to the results reported in [18], where an accuracy better than 10% was found for lesions larger than 4 mL. The authors used simulated data and phantom measurements as ground truth and compared three different correction methods. However, all three investigated methods require a precise knowledge of the true point spread function (PSF) of the tomograph, while in our approach only a rough estimate of the PSF is needed. In [15] accurate correction capability is reported even for very small lesions (diameter 8 mm), but this method, too, requires a precise knowledge of the scanner’s PSF. We believe that requiring accurate knowledge of the PSF as a prerequisite is problematic and a potential source of substantial error of the partial volume correction, especially in a clinical context, where data sets might undergo individually different postprocessing/smoothing. Our approach, on the other hand is insensitive to variation of the actual PSF within a reasonable range of uncertainty which is an obvious advantage.Gallivan et al. [8] report on very good results without knowledge of the PSF, but only approximately spherical object with homogeneous tracer uptake were considered, which does not apply to the mostly irregular lesions observed in real patient data. Other authors have proposed to use anatomical information from high resolution CT or MRI (see e.g. [33-35]). This, however, requires very accurate coregistration of PET and CT/MRI which can be problematic even for modern PET/CT or PET/MRI systems due to patient motion during measurement. Probably more important, this approach rests on the assumption that the morphologically delineated lesion is identical to the hypermetabolic region observed in PET. As is well known, this assumption is by no means always correct. Such a lack of spatial concordance between morphological and functional signal would in turn lead to uncontrollable errors of the PVE correction. In this respect correction procedures relying exclusively on analysis of the PET data alone seem preferable.We, therefore, believe that the proposed method represents a viable, partly superior, alternative to other methods already discussed in the literature.

## Conclusion

The presented approach to partial volume correction using local background determination distinctly improves quantitative accuracy of the correction in comparison to similar, previously described model-free approaches relying on a homogeneous background for the whole lesion. The improvement is especially pronounced for small lesions where the correction becomes numerically large. We conclude that adequate consideration of background heterogeneities on a per-voxel basis is mandatory to achieve reliable partial volume correction.

## Competing interests

The authors declare that they have no competing interests.

## Authors’ contributions

FH developed and implemented the PVC algorithm, performed part of the data analysis and is the main author of the manuscript. JL and JP performed part of the data analysis. BBB and LO selected the patient studies and performed the lesion delineation in the original patient data. JS and JK provided intellectual input and reviewed the manuscript. JVDH had the initial idea for the voxel based PVC and wrote part of the manuscript. All authors read and approved the final manuscript.
